# Laparoscopy-assisted posterior low anterior resection of rectal cancer

**DOI:** 10.1186/1471-230X-14-158

**Published:** 2014-09-12

**Authors:** Hao Qu, Yan-Fu Du, Min-Zhe Li, Yu-Dong Zhang, Jian Shen

**Affiliations:** Department of General Surgery, Beijing Chaoyang Hospital, Capital Medical University, Beijing, 100020 China

**Keywords:** Laparoscope, Rectal cancer, Posterior low anterior resection, Anal functional recovery

## Abstract

**Background:**

Laparoscopy-assisted low anterior resection (LAR) of colorectal cancer, using a posterior surgical approach, is a difficult and controversial procedure to perform. We report successful operations on 13 patients with clear surgical margins and no serious complications.

**Methods:**

Thirteen patients [10 males and three females, age range: 48 to 69 years (median: 61 years)] with low adenocarcinoma confirmed by preoperative colonoscopic biopsy (four stage T1; nine stage T2) were resected. The distance from inferior edge of tumor to dentate line was 2 ~ 5 cm (average: 3.4 cm). Intraperitoneal laparoscopy was performed to isolate rectosigmoid and mesocolon moving toward distal end of the tumor. Perineal operation was performed in the prone clasp-knife position.

**Results:**

The circumferential resection margin (CRM) was negative in all cases. No serious postoperative complications occurred. There were four cases of perineal wound infection, two cases with superficial perineal wound dehiscence, and two cases with persistent postoperative sacral pain. All 13 patients passed the Wexner continence test and had satisfactory anal function during a mean 18-month postoperative follow-up period.

**Conclusion:**

Laparoscopic posterior LAR of colorectal cancer is a safe and reliable treatment for patients with low colorectal cancer, increasing the chance of anal functional recovery.

**Trial registration:**

Chinese Clinical Trial Register ChiCTR-ONC-14005145. Registered 19 August 2014.

## Background

Low rectal cancer is a relatively common malignant disease with high morbidity and mortality rates [1, 2]. Globally, low anterior resection has been the mainstay of surgical therapy for rectal cancer since the 1970’s. Despite the best efforts of experienced surgeons, 5-year survival rates have ranged from 27% to 42% [3]. This conventional technique has also been associated with a high risk of damage to the autonomic pelvic nerve plexus, resulting in sexual and bladder dysfunction [3].

The introduction of TME [4, 5], first described by Heald et al. in 1982 [6], was a milestone in the treatment of rectal cancer [7]. Many studies, however, have shown that TME is a technically demanding procedure that requires excision of the intact mesorectum, in the narrow space of the pelvic cavity [3, 8–10].

In addition to TME, rapid advancements in laparoscopic technique have allowed for the laparoscopic resection of rectal cancer [5, 11–14] with improved postoperative recovery and earlier return to full activity. However [5, 15–17], rectal cancer has been excluded from most trials of laparoscopic resection of the large bowel because of the technical complexity of the procedure [17–23]. The anatomic position of the rectum makes access more difficult, and TME, with preservation of the autonomic nerves and the sphincter apparatus (important to maintain bladder control, continence, and sexual function) [18, 24], is associated with a considerably higher rate of complications than that of colonic surgery. This is especially true if the surgeon does not have sufficient experience in open TME and advanced laparoscopic surgery, although the MRC CLASSIC trial has shown that the oncological outcomes are similar to open [25].

In addition, for middle and low rectal cancer, especially in overweight patients or patients with a narrow pelvis, it is difficult to treat the bowel at the distal end of a tumor. Thus, for middle and low rectal cancer, especially for patients with a lower tumor edge at 2–5 cm from the dentate line, laparoscopy-assisted posterior low anterior resection (LAR) of rectal cancers is performed, although the procedure remains controversial. T3/T4 tumors would still be dealt with in the normal way.

Because of these issues, we reported our surgical experience with laparoscopy-assisted posterior LAR of rectal cancer cases that had a tumor edge within 2 to 5 cm from the dentate line. The term "posterior LAR" refers to the low anterior resection for rectal carcinoma completed via the posterior perineum. The term "posterior" (i.e., posterior approach) refers to the approach of posterior perineoplasty.

## Methods

### Clinical data

This prospective study was approved by the Medical Ethics Committee of Beijing Chaoyang Hospital, Capital Medical University, Beijing and the written informed consent for participation in the study was obtained from participants.

Patients were included discontinuously from September 2009 to February 2012. During this period, a total of 223 cases received rectal cancer surgery, 96 cases received laparotomy, and 127 cases received laparoscopic surgery in our hospital, of which 13 cases were reported. From June 2011 to June 2013, all 13 cases of rectal cancer were treated with laparoscopy-assisted posterior LAR in the Department of General Surgery of Beijing Chaoyang Hospital. These 13 patients were specifically recruited to test the technique of laparoscopic-assisted middle and low rectal cancer surgery. This technique is especially suited for patients who have large body habitus and/or contracted pelvis. In these cases, it is very difficult to separate distal bowel from tumor and complications such as presacral venous bleeding or bowel rupture can occur during the process of bowel separation and exposure, and sometimes the distance from the dentate line to the lower tumor edge does not reach 2 cm. During intraperitoneal surgery, when the separation reaches the pelvic floor, the patients are turned to the inverted ‘V’ or prone clasp-knife position position, and through posterior perineal approach, the distal bowel involved with tumor can be resected under direct visualization to further complete resection and anastomosis, which improves the safety and reliability of the procedure.

The patient group included 10 males and three females ranging in age from 48 to 69 years (median age: 61 years). The lower tumor edge was 2–7 cm from the dentate line (average distance: 3.4 cm) in all patients. After the anastomosis, if the anastomotic stoma was within 2–3 cm from the dentate line, we performed a protective stoma, but if the anastomotic stoma was more than 3 cm from the dentate line, we did not perform a protective stoma. Grading was based on preoperative MRI examination and included four cases with stage T1 cancer and nine cases with stage T2 cancer. Because patients were all diagnosed as T1 or T2 stage colon cancer before surgery, additional adjuvant chemotherapy was not given before the surgery. The reason we selected middle and low rectal cancer patients at the preoperative T1 and T2 stages was that preoperative neoadjuvant chemoradiotherapy could be shelved temporarily in these patients, as the preoperative neoadjuvant chemoradiotherapy might have an effect on the healing of the posterior perineal wound.

Preoperative laboratory evaluation was normal and preoperative colonoscopic pathology confirmed tumor histology as adenocarcinoma in all cases (Table 1).

**Table 1:** **Patients’ clinical data**

Case #	Preop staging	Distance from lower tumor edge to dentate line (cm)	End ileostomy	Preop Wexner	Postop Wexner
1	T2	3.5	N	0	0
2	T2	4	N	0	4
3	T2	4.5	N	0	4
4	T1	3	Y	0	0
5	T2	3	Y	2	5
6	T2	4.5	N	0	3
7	T1	2.5	Y	4	6
8	T2	4	N	0	5
9	T2	2.5	Y	0	2
10	T2	2.5	Y	1	N/A
11	T1	3	Y	0	N/A
12	T1	3.5	N	0	N/A
13	T2	4	N	0	N/A

*N*/*A*: not available.

Surgical inclusion criteria were: 1) low colorectal cancer with a distance from the inferior edge of the tumor to the dentate line ranging from 2 to 7 cm; 2) preoperative MRI staging from T1 ~ T2N0M0. Exclusion criteria were: 1) severe cardiovascular, pulmonary or cardiac dysfunction; 2) history of tuberculous peritonitis; 3) hematologic disorder or coagulopathy.

### Surgical procedure

Patients were placed in the straddle position with their head bent 30 degrees downward and to the right. Abdominal laparoscopic surgery followed total mesorectal excision (TME) principles, [6, 24, 26, 27] i.e., the first half of the posterior operation followed the surgical principles of TME, and after completing the resection, this surgical approach was not applied until cutting off the distal bowel.

A total of five trocars were needed. One was inserted 1 cm above the navel as the observation port for the endoscope. The main operative port was in the right lower abdomen and additional operative ports sites were located to the right and left of the navel and left lower abdomen.

**Figure 1:**
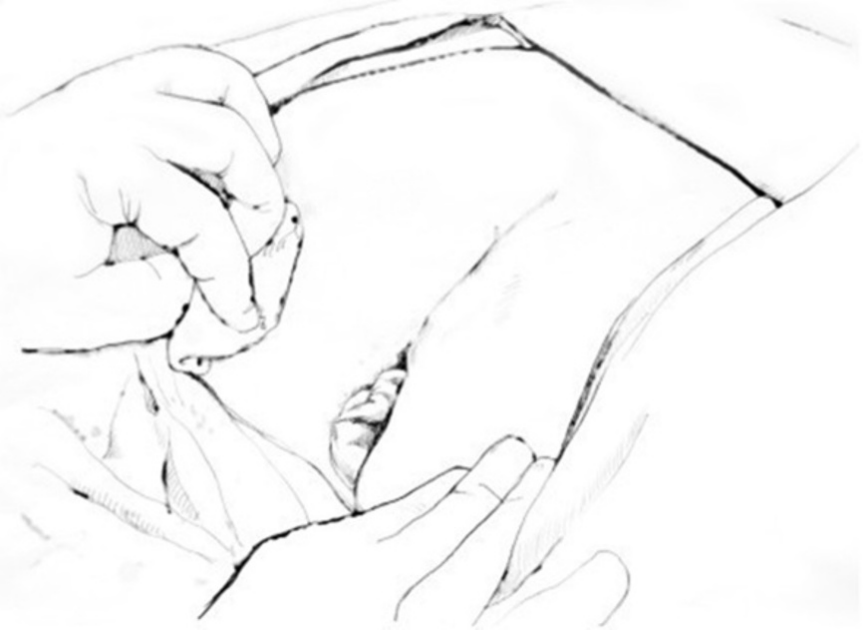
**Posterior perineal incision.**

**Figure 2:**
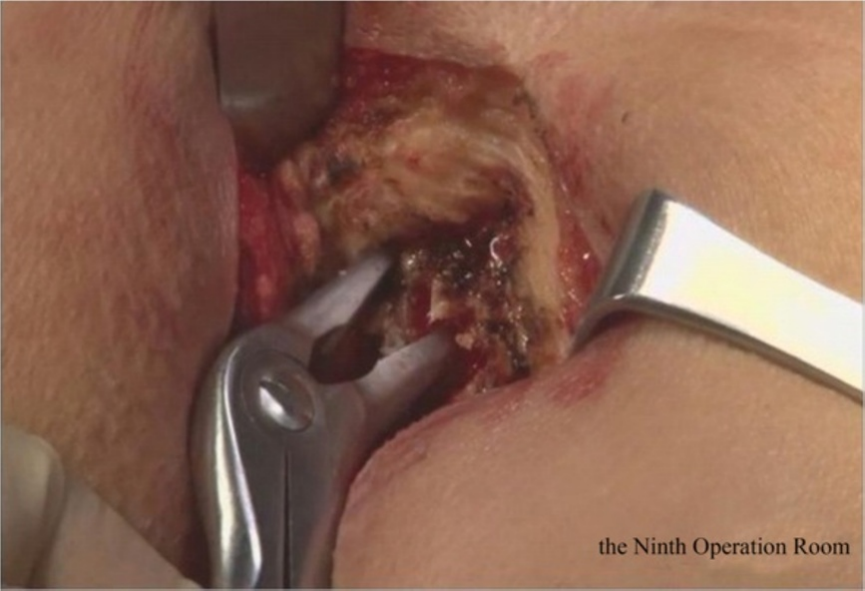
**Removal of the sacrococcyx.**

**Figure 3:**
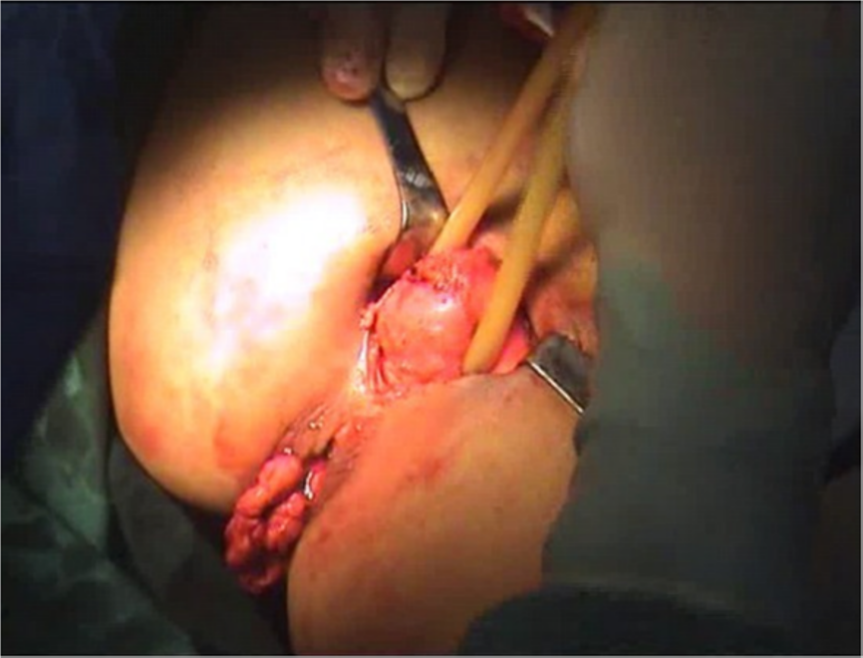
**Separated bowel lifted out of body.**

**Figure 4:**
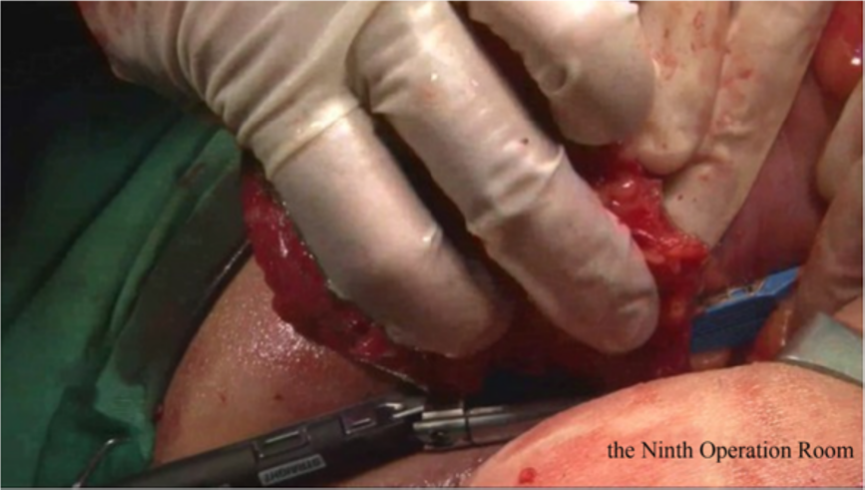
**Resection of distal rectum.**

**Figure 5:**
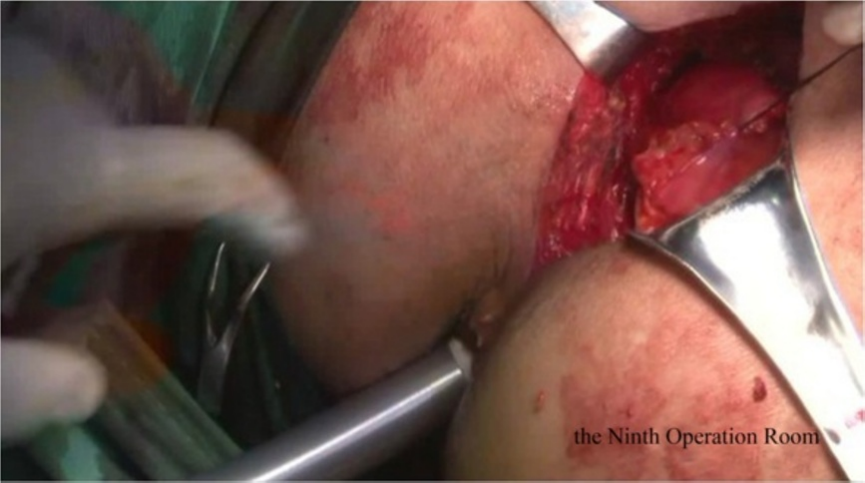
**Anastomosis of colon and rectum.**

**Figure 6:**
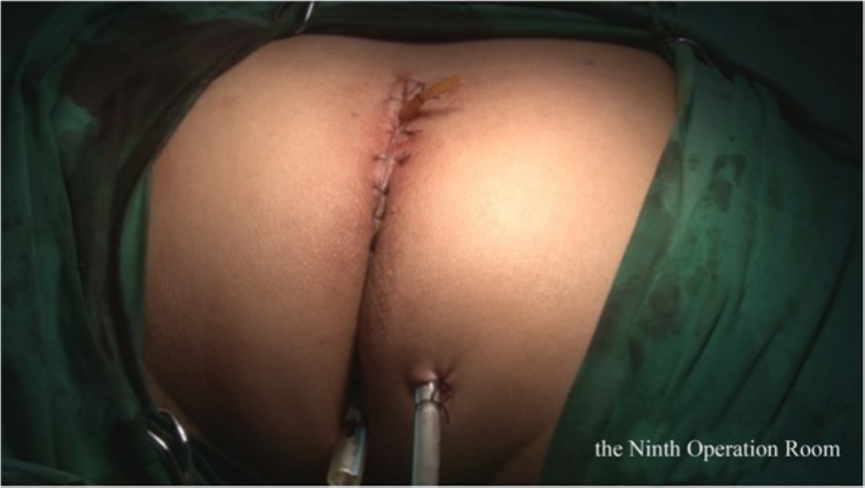
**Placement of presacral drainage tube**, **wound drainage and anal canal.**

Briefly, the right side of the sigmoid mesocolon was separated, the accompanying veins of the inferior mesenteric artery were exposed by dissection, the vascular roots of the lymph nodes were cleaned, and the rectal artery and its accompanying veins were cut. In order not to affect circulation of the intestinal loop at the proximal end, the left colic artery was retained [28]. Along the gap between the rectal inherent fascia and pelvic wall fascia, the tip of the coccyx was separated by sharp dissection. The peritoneal wall of the rectum was incised and folded back. Separation of the anterior wall of the rectum from the seminal vesicles in males (or from the vagina in females) was performed in the gap between Denonvillier’s fascia. The bilateral ligaments were cut off, with care taken to protect the pelvic autonomic nerve. Once the dissociation reached the distal end of the tumor, gauze was placed in front of the sacrum to mark the location.The patient was then turned over to the prone clasp-knife position with legs apart. The surgeon stood between the legs, and the assistant stood outside the legs. A straight incision was made from 2 cm above the coccyx to 1 cm above the posterior edge of the anus (Figure 1).Skin and subcutaneous tissue layers were successively incised, and the coccyx and the fifth sacrum were removed (Figure 2).Waldeyer’s fascia, iliac coccygeal muscle, pubococcygeus muscle, pelvic diaphragm, and superior and inferior fascia were longitudinally incised to find the presacral gauze, and the free rectosigmoid and mesocolon (which were isolated laparoscopically) were lifted out of body through the incision (Figure 3).The bowel at the distal end of the tumor was separated and exposed under direct visual inspection by cutting the distal rectum 2 cm away from the lower edge of the tumor (Figure 4).The proximal bowel and its mesentery, including the mesenteric vascular bed, were removed through the posterior perineal incision. The sigmoid mesocolon was sutured and fixed at the posterior midline to prevent torsion during anastomosis. The mesentery was treated and the proximal bowel was cut, leaving a sufficient length. Anastomosis of the rectosigmoid was performed from the anus under direct visual inspection using the circular stapler (Figure 5).A presacral drainage tube was placed from the perineum and an additional drainage canal was placed in the anus (Figure 6).

The puborectalis and coccygeal rectus were successively sutured. A subcutaneous drainage tube was placed and subcutaneous tissue and skin were sutured.

For the six patients with a lower tumor edge approximately 2–3 cm from the dentate line, additional protective end ileostomy was performed, and ileostomy reversal surgery was performed three months after the operation.

All the patients were evaluated with preoperative Wexner continence scoring [29], which was repeated at 1 year after the surgery or at 1 year after stoma reversal surgery. Anal manometry was performed before stoma reversal surgery in the six patients who underwent end ileostomy.

## Results

All surgery was performed to achieve radical resection, and the mean operative time was 224 mins (range: 190–245 mins). Mean operative blood loss was 95 mL (range: 70–165 mL). Postoperative drainage tubes were removed at an average of 5.8 days (range: 4–8 days) after surgery. No deaths occurred in this patient group. Postoperative pathology revealed two cases of T1N0M0, 10 cases of T2N0M0, and one case of T2N1M0. The circumferential resection margin (CRM), which included the distal margins, was negative in all cases.

No serious postoperative complications (including anastomotic leaks or strictures) were encountered. Five patients had minor postoperative complications, including two cases of perineal wound infection, one case of partial superficial dehiscence of the perineal wound, and two cases of persistent postoperative sacrococcygeal pain. Of the two cases of perineal wound infection, one case had infection of the protective ileostomy and the other of non-protective ileostomy. The patient with partial superficial wound dehiscence had undergone non-protective ileostomy. Dressings were changed as part of the treatment for wound infection and partial dehiscence. Symptoms of persistent sacrococcygeal pain were alleviated with oral non-steroidal anti-inflammatory agents for 2 weeks, and patients’ symptoms eased after 1 month. All complications occurred within 30 days, and we did an active follow-up on all patients.

The average follow-up time was 18 mos (range: 5–29 mos). In the six patients with end ileostomy, an ileostomy reversal operation was completed 3 months after the first surgery. Anal manometry was performed before the second surgery if maximum anal resting and squeeze pressures were normal. Wexner continence scoring showed a preoperative median score of 0 (range: 0 to 4, n = 13) and a postoperative median score of 4 (range: 0 to 6, n = 9). Postoperative defecation (either solid or liquid) was well-controlled. The median number of postoperative bowel movements was three per day (range: one to seven per day). There was no report of urgent defecation and all patients were satisfied with their postoperative anal function. No cases of tumor recurrence were found during the short-term follow-up period after surgery.

## Discussion

We performed successful laparoscopy-assisted low anterior resection (lap-LAR) of colorectal cancer, using a posterior surgical approach, on 13 patients. All 13 patients had satisfactory anal functional recovery. In comparison with previous studies which described lap-LAR without the posterior approach [5, 8, 13, 16, 18, 30–32], we found no serious complications when performing Lap-LAR using the posterior approach and the CRM was negative in all 13 cases.

Lap-LAR of the rectum in cases of middle and low rectal cancer, without the posterior approach, can be technically challenging. Difficultly in grasping the distal resection margin, sawtooth-like breaks occurring from repeated cutting of closures (which increases the chance of leakage from the break), and equipment costs are just a few of the drawbacks [33]. The distal bowel wall is also difficult to handle in patients who are overweight, and in cases of hypertrophy of bowel and mesentery. The relatively narrow space in small pelves (including the inherent narrow pelves in male patients), and the added difficulty of using operating instruments under laparoscopy add to the inherent risks. These issues often lead to difficulties in tumor distal rectum separation, exposure, and closure. It also can be especially difficult to achieve the minimum 2 cm bowel resection distance. Insufficient resection distance results in positive CRM. An inadequately closed distal bowel can also lead to weak closure or tears, which increase the chance of postoperative leakage. Additionally, relatively sharp instruments in a narrow pelvic area can damage blood vessels and cause bleeding, such as damage to the pudendal vein and presacral bleeding. We encountered three cases of pudendal vein bleeding in previous middle and low rectal cancer surgery. Lap-LAR of rectal cancer via a posterior approach, in cases of middle and low rectal carcinoma, was designed to overcome these difficulties.

In the 1970’s, Mason reported on a surgical approach for local resection of early low rectal cancer or villous adenoma through a posterior perineal pathway, in which the coccyx and the fourth or fifth sacrum was incised, or the Waldeyer’s fascia, anal sphincter, pelvic floor muscles, rectal inherent fascia, and rectal wall were cut open [34–37]. After resection of the local lesion, each layer was successively sutured and anal function was not affected after surgery. Qiu et al. [38–41] and Lin et al. [42] stated that this approach could provide a larger operating space and maintain anal function to the maximum extent, where conditions allowed. The difference between laparoscopy-assisted posterior LAR of rectal cancer and Mason’s operative approach is that after the peripheral longitudinal incision of Waldeyer’s fascia and the pelvic floor muscle, and entry into the laparoscopic pelvic canal, further surgery thereafter does not need to cut the pelvic floor muscle or the attachment site for the anal sphincter at the anal canal. Therefore, the injuries are minor, and, theoretically, the effect on anal function is less.

Recently, several attempts have been made to improve proper handling of the distal bowel to retain anal function to the maximum extent in cases of middle and low rectal cancer. The Anterior Perineal Plan E for Ultra-low Anterior Resection of the Rectum (APPEAR) technique [32, 43, 44], involved gaining access through the rectum and vagina/prostate plane to the perineal body, then accessing the small pelvic canal, performing joined abdominal operations, and completing the resection and anastomosis. With patients in the lithotomy position, there was no need to turn the patient over to the clasp-knife position, and it was also conducive to retaining anal sphincter function. Limbert and Almeida reported on middle and low rectal cancer resection and anastomosis through anoscopy [33]. Fukunaga et al. [45] used the traditional straight-line cutting closure device to produce a pubic symphyseal incision to finish processing the distal rectum.

Compared with these methods, lap-LAR of rectal cancer, using a posterior approach, has the advantages of a simple posterior perineal operation, shallow surgical view, clear anatomy field, minor bleeding, and minimal effects on anal function. This procedure also maximizes the advantages of the laparoscopic surgical technique. If a larger operating space is needed when handling the low pelvic position, incision of the posterior perineal wound makes the surgical view shallow and spacious which is conducive to the use of surgical equipment. When handing the resection of the bowel under direct visual inspection, it is easy to master the standard of bowel removal at least 2 cm beyond the tumor distal end [46, 47], therefore, it is possible to retain anal function to the maximum extent (if the patient’s condition permits). Laparoscopy-assisted posterior LAR of rectal cancer not only can conveniently handle the distal bowel of mid-low rectal cancer, but also can handle certain pelvic floor complications which appear when separating bowel under endoscopy, such as bleeding. In addition, the procedure is suitable for rectal cancer patients who have invasion of the prostate or vaginal wall, it is convenient for anastomosis, and there is minor postoperative abdominal pain. It also facilitates the placement of drainage tubes from the perineum. In addition, the possibility of perineum wound recurrence is not increased.

Our study had several limitations. Since our results represent only the early stage of our research, the number of cases was limited and the follow-up period was short. In addition, this surgical approach applies only to mid-low position rectal cancer and, therefore, its indications are limited as it does not apply to all patients with rectal cancer. This technique, however, is useful when presented with the difficult condition of handling a distal bowel involved with tumor.

Our surgical technique, itself, had several disadvantages, primarily involving placement of the posterior perineal incision close to the anus. This placement exposed the wound to a contaminating area. Additionally, anastomosis and other operations were completed via the anus, increasing the infection risk. In our patient group, there were two cases of posterior perineal incision infection, including one case with protective stoma and the other with non-protective stoma. Therefore, additional care is necessary to improve surgical isolation to prevent contamination. In our patients, there was also one case of partial wound dehiscence in a non-protective stoma patient where the dehiscence occurred during early postoperative defecation in the sitting position. Considering that defecation in the squatting or sitting position can increase perineal wound tension, patients may need to defecate in a supine or semi-recumbent posture in the early postoperative period to prevent constipation and maintain a smooth bowel movement. There were two instances of postoperative sacrococcygeal pain and discomfort. After resection of the sacrococcyx, there is a strong mechanical force of sharp bone edge against local soft tissue which takes time to heal. Oral non-steroidal anti-inflammatory agents can alleviate pain and speed up the healing process.

A further disadvantage of our technique relates to the need to change the patient’s position during surgery from the straddle to the leg-separation clasp-knife position under general anesthesia. The cumbersome process of changing the patient’s position requires a concerted effort on the part of the surgeons, anesthesiologists, and nurses and it increases the total operating time. Wang et al. [48] and Han et al. [49] have reported on their attempts to avoid switching to the leg-separation clasp-knife position for rectal cancer patients who needed cylindrical abdominoperineal resection surgery.

Most studies of low rectal cancer surgeries have focused on the preservation of the anal sphincter while maintaining oncologic safety. In laparoscopic low rectal cancer surgeries, the technique is particularly demanding with regard to sphincter preservation. Application of the linear staple device to the distal rectal tube, for example, presents challenges in obtaining safe margins. Low rectal cancer surgery is especially difficult in terms of preservation of sphincter function because of limitations in applying the double-stapling technique [1, 50]. Transabdominal transanal resection (TATAR) with total mesorectal excision (TME) by laparoscopy tends to lessen the technical difficulties encountered by many surgeons, according to recent reports [1, 51]. However, using our posterior approach and stapling technique, all 13 patients showed satisfactory anal functional recovery during the early postoperative and follow-up period, as measured by Wexner continence scoring.

## Conclusion

Laparoscopy-assisted posterior LAR of rectal cancer is a safe and reliable technique which increases the chance of anal functional recovery for patients with difficult-to-handle middle and low rectal cancers.
